# Safety of bloodless autologous stem cell transplantation in Jehovah's Witness patients

**DOI:** 10.1038/s41409-019-0777-9

**Published:** 2020-01-02

**Authors:** Alyssa Beck, Robert Lin, Ali Reza Rejali, Muni Rubens, Ronald Paquette, Robert Vescio, Noah Merin, Margarita Guerrero, Yvette Federizo, Michelle Lua, Leticia Uy, Lorraine Hernandez, Mohana Allred, Ronald Legaspi, Melissa Leaverton, Sara Oliva, Rhona Castillo, Lorna Dean, Jennifer Bourke, Sara Cooper, Seda Gharapetian, Jose Causin, Christopher Lopiccolo, Laura Ann Snoussi, Patricia VanStrien, Michael Lill, Yuliya P. Linhares

**Affiliations:** 1 Department of Internal Medicine, Cedars-Sinai Medical Center, 8700 Beverly Boulevard, Los Angeles, CA 90048 USA; 2 Department of Pharmacy, Cedars-Sinai Medical Center, 8700 Beverly Boulevard, Los Angeles, CA 90048 USA; 3 Miami Cancer Institute, Baptist Health South Florida, 8900 North Kendall Drive, Miami, FL 33176 USA; 4 Department of Hematology/Oncology, Cedars-Sinai Medical Center, Samuel Oschin Comprehensive Cancer Institute, 8700 Beverly Boulevard, Los Angeles, CA 90048 USA

**Keywords:** Stem-cell research, Epidemiology

## Abstract

Due to the curative potential and improvement in progression-free survival (PFS), high-dose chemotherapy followed by autologous stem cell transplantation (ASCT) is considered the standard of care for several hematologic malignancies, such as multiple myeloma, and lymphomas. ASCT typically involves support with blood product transfusion. Thus, difficulties arise when Jehovah’s Witness patients refuse blood transfusions. In order to demonstrate the safety of performing “bloodless” ASCT (BL-ASCT), we performed a retrospective analysis of 66 Jehovah's Witnesses patients who underwent BL-ASCT and 1114 non-Jehovah’s Witness patients who underwent transfusion-supported ASCT (TF-ASCT) at Cedars-Sinai Medical Center between January 2000 and September 2018. Survival was compared between the two groups. Transplant-related complications, mortality, engraftment time, length of hospital stay, and number of ICU transfers were characterized for the BL-ASCT group. One year survival was found to be 87.9% for both groups (*P* = 0.92). In the BL-ASCT group, there was one death prior to the 30 days post transplant due to CNS hemorrhage, and one death prior to 100 days due to sepsis. Based on our data, BL-ASCT can be safely performed with appropriate supportive measures, and we encourage community oncologists to promptly refer JW patients for transplant evaluation when ASCT is indicated.

## Introduction

High-dose chemotherapy (HDT) followed by autologous stem cell transplantation (ASCT) is the standard of care for patients newly diagnosed with multiple myeloma due to the improvement in progression-free survival (PFS) with this approach [1, 2]. ASCT for multiple myeloma patients beyond first remission may offer a survival benefit [3–5]. HDT followed by ASCT is a standard of care for relapsed diffuse large B cell lymphoma as well as multiple other lymphoma subtypes in different stages of treatment [1, 6]. In the course of ASCT it is customary to provide red cell and platelet transfusion support on a preventative basis. Most institutions recommend red cell transfusion for hemoglobin levels < 7 g/dL, and platelet transfusion for platelet counts < 10 × 10^9^/L. On average, an ASCT recipient receives 1–2 units of PRBCs and 2 units of platelet transfusions in the posttransplant course [7, 8]. Platelet and red cell posttransplant transfusion requirement may depend on institutional protocol, pretransplant hemoglobin and platelet counts [9, 10]. Thus, given that ASCT is the standard of therapy for several malignancies, and given that it typically involves blood product transfusions, difficulties arise when a patient refuses blood transfusions.

In particular, the Jehovah’s Witness (JW) faith is one of the more well-known religious groups which decline blood transfusions. The JW organization, the Watchtower Society, mandated the policy on refusal of blood transfusion in 1945 based on the following quote in the Bible, Leviticus 17:12 “None of you may eat blood, nor may any foreigner living among you eat blood” [11]. The ones that do choose to accept blood and platelet transfusions may be excluded from their religious community based on the following Biblical quote, Leviticus 17:10, “If anyone from the house of Israel or foreigner living among them eats any blood, I will set My face against that person and cut him off from among his people.” JW who choose to accept blood may be subject to “disfellowship” from the JW organization as well as shunning and even isolation from their family and friends. Repentance and humility to the degree satisfactory to the JW elders may lead to the member “reinstatement” [12]. Due to these beliefs, JW patients are placed in a difficult position where they must choose to either receive standard medical care and get cast out from their religious community or choose to receive suboptimal care and remain within their religious community.

In addition, many JW patients with myeloma/lymphoma/leukemia had to forego ASCT when it is indicated due to the fact that most transplant centers do not offer stem cell transplant treatment without transfusion support. This may be due to perceived risks of increased risk of adverse events, such as bleeding and cardiovascular complications, and/or due to the lack of experience with bloodless transplants. Nevertheless, there is now a growing body of evidence that with appropriate supportive care, bloodless autologous stem cell transplants (BL-ASCT) can be as safe and effective as ASCT with transfusion support (TS-ASCT) [7, 8, 13, 14]. In this study we describe our center’s experience with 70 BL-ASCT cases with regards to their engraftment outcomes, complications, mortality, and survival data.

## Materials and methods

We reviewed electronic medical records of all patients who underwent BL-ASCT at our institution between January 2000 and September 2018. In order to obtain the comparator data for the OS, we reviewed contemporaneously matched medical records of the patients who underwent TS-ASCT for the same malignancy indications during the same time period. A total of 1180 patients, 66 (5.6%) undergoing BL-ASCT and 1114 (94.4%) undergoing TS-ASCT were included in the study. The study was approved by the Institutional Review Board of the Cedars-Sinai Medical Center, Los Angeles, USA.

Patients with a malignancy indication for ASCT and adequate organ function were selected for autologous transplantation. Refusal of platelet and red cell transfusions did not exclude patients from ASCT. Candidates for BL-ASCT did not have to meet additional stem cell transplant eligibility criteria compared with the patients undergoing TS-ASCT. It was preferable for the patients to have pretransplant hemoglobin of 10 g/dL and platelet count of equal or over 100 × 10^9^/L. There was no standardized protocol for the achievement of the desired hemoglobin and platelet thresholds pre-BL-ASCT.

Patients who met institutional criteria for ASCT underwent standardized granulocyte colony-stimulating factor (G-CSF) mobilization and apheresis for collection of stem cells. Patients were admitted prior to stem cell transplantation and given one of several conditioning regimens, including: melphalan, BEAM, R-BEAM, carboplatin + etoposide, carboplatin + etoposide + thiotepa, total body irradiation + cyclophosphamide, total body irradiation + cyclophosphamide + etoposide, busulfan + cyclophosphamide, carmustine + thiotepa.

Autologous stem cells were infused on day 0. During their hospital stay, all patients received standard prophylactic acyclovir/valacyclovir, levofloxacin,“-azole” antifungals, in addition to G-CSF (starting day +5).

### Supportive care

A variety of measures were taken to minimize blood loss, decrease bleeding risk, and stimulate hematopoiesis for all patients (Table 1). These interventions include but are not limited to minimizing blood loss via collection of routine labs every other day and pediatric tube use, intravenous or oral iron supplementation, vitamin K supplementation, folic acid supplementation, erythropoietin, antiemetics, proton pump inhibitors, stool softeners, nasal saline spray, aminocaproic acid, strict bed rest under certain conditions, and (for female patients) oral contraceptives.

**Table 1:** Supportive care measures for Jehovah’s Witnesses undergoing HSCT.

Universal measures	
	Minimize phlebotomy (draw labs every other day in pediatric tubes, avoid unnecessary tests)
	Vitamin K 10 mg PO TIW, folic acid 1 mg PO TID, vitamin B12 1 g PO daily
	Stool softeners (e.g. docusate 250 mg PO BID)
	Aggressive antiemetic pharmacotherapy
	Proton pump inhibitor (e.g., nexium 40 mg PO daily)
	Nasal saline spray
	Ferrous sulfate 325 PO TID with ascorbic acid 100 mg PO TID^a^
	Fall and bleeding precautions
	Cessation of menses (e.g. medroxyprogesterone 10 mg daily)
	No NSAIDs or aspirin
	G-CSF following completion of chemotherapy and transplant
	Aggressive treatment of fever with acetaminophen
	Avoid myelosuppressive antibiotics (e.g., bactrim, linezolid)
	Erythropoietin 150 units/kg SQ TIW starting Day +1 to keep Hgb > 11 g/dL. May increase to 150 units/kg SQ daily
**Anemia—Hgb less than**	
10 g/dL	Iron sucrose 100 mg IV weekly for 4 weeks (if not already on and in place of oral iron)
6 g/dL	Bed rest
	Oxygen 2 L/min via nasal cannula
	Bedside commode with assistance
	Vitals q4 h
	Initiate "fall precautions" including bed alarm
4 g/dL	Absolute bed rest
	Bedpan and urinal in bed only (no commode or bathroom use)
	Oxygen via nonrebreather at all times
	Minimize coughing, retching, vomiting via optimal medical management
**Thrombocytopenia—platelets less than**	
50 × 10^9^/L with bleeding	Start aminocaproic acid 1–4 g PO/IVPB q4–6 h, adjust dose until bleeding resolves
	DDAVP 0.3 mcg/kg IVPB q12 h for 36 h if bleeding persists on aminocaproic acid
	Nasal vasoconstrictors for epistaxis (e.g., neosynephrine)
	Conjugated estrogens PRN for vaginal bleeding (e.g., Premarin 25 mg IVP)
10 × 10^9^/L	Start low-dose aminocaproic acid 1–4 g PO/IVPB q4–6 h
	Obtain urinalysis prior to starting aminocaproic acid (assess for hematuria)
	Vitamin K 5–10 mg IVPB daily
	Stop all anticoagulation
5 × 10^9^/L	Topical aminocaproic acid if patient has mucositis

*HSCT* hematopoietic stem cell transplant, *TIW* three times weekly, *DDAVP* desmopressin, *G-CSF* granulocyte colony-stimulating factor (e.g., filgrastim), *NSAID* nonsteroidal anti-inflammatory drug

^a^Changed in June 2013 to iron sucrose 100 mg IV weekly as a universal measure regardless of Hgb to avoid risk of GI bleed with oral iron supplementation

### Statistical analysis

Neutrophil engraftment was defined as the first day of absolute neutrophil count ≥ 0.5 × 10^9^/L for 3 consecutive days. Platelet engraftment is the first day of platelets ≥ 20 × 10^9^/L for 3 consecutive days. Days were calculated with respect to day of hematopoietic stem cell infusion, which is designated as day 0. Patients who passed away due to transplantation-related mortality (TRM) before engraftment were censored at the date of their death. Posttransplant overall survival (OS) was defined from the date of transplantation to the date of death or last follow-up. OS was estimated using the Kaplan–Meier method and was compared using the log-rank test.

Kaplan–Meier was also used to determine median follow-up post transplant. Nominal data were compared using Chi-square or Fisher exact test, while ordinal data were compared using the Mann–Whitney test. All statistical analyses were performed using GraphPad Prism version 8.1.2 for Windows, GraphPad Software, La Jolla California USA, www.graphpad.com.

We performed a limited analysis of the TS-ASCT data for the purposes of comparing OS between the BL-ASCT and TS-ASCT patients. We included only first transplant events for the OS calculations.

## Results

### Patient characteristics

Patient characteristics are summarized in Table 2. There were 70 BL-ASCT transplants performed from June 2000 to December 2018. Diagnoses included multiple myeloma (*n* = 31), Non-Hodgkin lymphoma (*n* = 17), Hodgkin lymphoma (*n* = 13), ALL (*n* = 2), AML (*n* = 1), and germ cell tumor (*n* = 2). Four of the seventy transplant cases were second BL-ASCT cases with the first BL-ASCT performed at Cedars-Sinai and included in this analysis. A total of 66 patients received BL-ASCT. The median age at the time of first transplantation was 53 years (range 17–72 years old) in the BL-ASCT.

**Table 2:** Patient characteristics.

	BL-ASCT	TS-ASCT	*p*
Total number of patients	66	1114	
Total number of transplants	70	1212	
Age at 1st transplant, years			
Median	53	56	0.03
Range	17–72	17–84	
Sex			
Male	31 (47%)	663 (60%)	0.04
Female	35 (53%)	451 (40%)	
Ethnicity			
Caucasian	24 (36%)	686 (62%)	0.12
Hispanic	22 (33%)	221 (20%)	
African American	15 (23%)	70 (6%)	
Asian	4 (6%)	99 (9%)	
Other	1 (2%)	38 (3%)	
Diagnosis			
Multiple myeloma	31 (47%)	646 (58%)	0.12
Non-Hodgkin lymphoma	17 (26%)	310 (28%)	
Hodgkin lymphoma	13 (19%)	106 (10%)	
Testicular germ cell tumor	2 (3%)	25 (2%)	
Acute myelogenous leukemia	1 (2%)	27 (2%)	
Acute lymphoblastic leukemia	2 (3%)	0	
Median follow-up post transplant, months			
After 1st AUTO SCT	63	90	0.03

*p* values corresponds to Chi-square, Fisher exact, or Mann–Whitney test

We reviewed select data for TS-ASCTs performed from January 2000 to December 2018. We identified 1114 patients who underwent TS-ASCT for the indications matching BL-ASCT (multiple myeloma, lymphoma, germ cell tumor, acute lymphoblastic leukemia, or acute myeloid leukemia). Patients who underwent TS-ASCT were older than BL-ASCT patients with a median age of 56 years (range 17–84) at the time of first transplant.

### BL-ASCT engraftment data

According to the institutional standard operating procedure, the requested stem cell dose for one ASCT was at least 5 × 10^6^ CD34 cells/kg. A median of 6.4 × 10^6^ CD34+/kg cells were infused (range 1.1–27.5 × 10^6^ CD34 cells/kg). Few patients were unable to achieve the target collection, but were transplanted after the risk versus benefit discussion. Engraftment data are reported in Table 3. The average hemoglobin prior to conditioning therapy was 12.6 g/dL (range 9.4–15.4 g/dL). We evaluated hemoglobin, platelets and neutrophil engraftment up to day +30 post auto SCT. When the blood count data were not available on day +30, we used the blood count data from the day closest to day +30 (mean and median day +28, range 14–41 days). Patients experienced a median decrease in hemoglobin of 4.1 g/dL (range 1.4–4 g/dL). The median number of days with hemoglobin < 7 g/dL was 0 (mean 0.6, range 0–14). The median day +30 hemoglobin was 11.6 g/dL.

**Table 3:** Engraftment parameters for BL-ASCT patients.

	Median	Mean	Range
CD34 × 10^6^/kg infused	6.4	7.1	1.1–27.5
Pretransplant Hgb (g/dL)	12.6	12.6	9.4–15.4
Days to Hgb nadir	11	11.5	1–28
Days Hgb below 7 g/dL	0	0.6	0–14
Mean Hgb (g/dL)	10.5	10.6	7.6–14.8
Hgb nadir (g/dL)	8.5	8.7	5.4–14
Pretransplant PL (×10^9^/L)	189	200	71–478
Days to PL nadir	9	9	5–21
Days PL below 10 × 10^9^/L	3	3.7	0–17
Mean PL (×10^9^/L)	91	93	31–163
PL nadir (×10^9^/L)	4.5	6.5	1–38
Day +30 Hgb (g/dL)	11.6	11.4	5.9–15
Day +30 PL (×10^9^/L)	208	202	5–554
Time to NF engraftment (days)	12	11.9	9–17
Time to PL engraftment (days)	13	14.5	9–38
Duration of grade 3–4 anemia (days)	0	1.8	0–19
Duration of Gr 3 TCP (days)	9	10.1	0–34
Duration of Gr 4 TCP (days)	5	6.5	0–29

*PL* platelet, *NF* neutrophil, *TCP* thrombocytopenia

There was platelet decrease in all patients. Average platelet count prior to conditioning therapy was 199.7 × 10^9^/L (range 71–478 × 10^9^/L). We evaluated platelet counts up to day +30 post-SCT. The median decrease in platelets was 185 × 10^9^/L (range 70–440 × 10^9^/L). Median platelet count was 91 × 10^9^/L (range 31–163 × 10^9^/L). Median platelet nadir was 4.5 × 10^9^/L (range 1–38 × 10^9^/L). The median days to platelet nadir was 9 days (range 5–21 days). Patients experienced platelet engraftment after a median of 13 days (range 9–38 days). On day +30, the median platelet count was 208 × 10^9^/L (range 5–554 × 10^9^/L). CTCAE 5.0 Grade 4 thrombocytopenia lasted a median of 5 days (range 0–29 days). The median number of days with platelet count <10 × 10^9^/L was 3 (range 0–17 × 10^9^/L). One patient with AML engrafted neutrophils, but failed to engraft platelets by day +21 and was given a second stem cell infusion on day +24 with subsequent platelet engraftment on day +38. The only patients who did not engraft platelets were the two patients who experienced TRM prior to day +100.

All patients, except for the two patients who died prior to day +100 experienced neutrophil engraftment. The average number of days to neutrophil engraftment was 12 (range 9–17 days).

### BL-ASCT cardiovascular complications

There were six cardiac complication events (8.6%) with three patients being transferred to ICU. No fatalities resulted from cardiovascular complications. Four patients experienced atrial fibrillation and two had episodes of SVT requiring adenosine without subsequent events. None of the patients experienced bradycardia or hypotension due to a cardiac event. None of the patients who experienced cardiac events had hemoglobin nadir below 7 g/dL. BL-ASCT mean hemoglobin nadir was identical at 8.7 g/dL in patients who experienced cardiac complications and those who did not. Hemoglobin range was 11.6–7.5 gm/dL in BL-ASCT patients who experienced cardiac complications. Hemoglobin range was 14–5.4 g/dL in BL-ASCT patients who did not experience cardiac complications.

### BL-ASCT bleeding complications

Bleeding complications were classified using the WHO grading system and summarized in Table 4. There were 12 bleeding episodes (17.1%).

**Table 4:** Post transplant complications in BL-ASCT group.

Age gender	Diagnosis	Conditioning regimen	Days in ICU	Bleeding WHO grade	PL Nadir × 10^9^/L	Hgb Nadir g/dL	Comments
5 F	MM	Mel200	0	≤2	38	12.8	Mild hematemesis
62 F	NHL	R-BEAM	0	≤2	2	8.3	Hematemesis
57 F	MM	Mel100	13	3	1	5.4	Cardiac tamponade, rectal bleeding, H1N1 influenza, ARDS, sepsis, and multiorgan failure
30 F	CNS GCT	Carbo/Thio/VP16	3	4	31	14	CNS bleed, died despite maximum supportive care
21 M	HL	BEAM	0	3	1	6.6	Lower GI bleeding
37 F	HL	BEAM	0	3	5	6.4	Vaginal bleeding, epistaxis, blood in sputum
64 M	NHL	R-BEAM	0	≤2	3	7.1	Hematuria
24 F	HL	BEAM	0	≤2	2	8.9	Vaginal spotting
17 F	NHL	BEAM	0	≤2	5	7.3	Gum bleeding
47 M	NHL	BEAM	0	≤2	2	7.2	Petechiae lower extremities
53 F	MM	Mel200	0	≤2	2	7.7	Mild epistaxis
52 F	MM	Mel200	0	≤2	9	8.6	Epistaxis
67 F	MM	Mel200	1		2	8.3	A fib
61 M	MM	Mel200	2		8	7.5	A fib w/RVR
66 F	NHL	R-BEAM	0		2	8.1	A fib w/RVR
60 M	NHL	BEAM	0		3	7.7	A fib
53 M	MM	Mel200	0		13	11.6	SVT, adenosine administered
57 M	MM	Mel200	4		5	9	SVT, HTN
61 F	ALL	TBI/Cy	0		4	10.4	Tonic–clonic seizure

*MM* multiple myeloma, *NHL* non-Hodgkin’s lymphoma, *HL* Hodgkin’s lymphoma, *CNS GCT* central nervous system germ cell tumor, *ALL* acute lymphoblastic lymphoma, *Mel* melphalan, *Carbo* carboplatin, *Thio* thiotepa, *VP16* etoposide, *R* rituximab, *ARDS* acute respiratory distress syndrome, *GI* gastrointestinal, *A fib* atrial fibrillation, *RVR* rapid ventricular response, *SVT* supraventricular tachycardia, *HTN* hypertension

There was one fatal bleeding complication secondary to a CNS hemorrhage in a 30-year-old female patient with central nervous system germ cell tumor with persistent disease in the pineal gland area prior to transplantation. CNS hemorrhage happened on day +3 post-BL-ASCT with the platelet nadir of 31 × 10^9^/L (average 91 × 10^9^/L). There were eight episodes (11.4%) of WHO grade 1–2 bleeding, including two genitourinary, two hematemesis, three epistaxis one of which co-existed with vaginal bleeding, and two mucocutaneous. These episodes did not warrant ICU transfers and resolved with appropriate medical management. There were two grade 3 lower GI bleeding episodes (2.9%), and one grade 3 vaginal/epistaxis bleeding episode, neither required ICU transfer due to the bleeding event. Of the 12 patients who experienced bleeding events of any grade, three patients had hemoglobin decline to below 7 g/dL, none had hemoglobin decline to below 5 g/dL. In 9 out of 12 patients with bleeding events (75%), platelet nadir was below 5 × 10^9^/L.

### Neurologic complications

There were no fatalities resulting from neurologic complications. One patient had a tonic–clonic seizure without any subsequent episodes. The patient did not require an ICU transfer.

### Hospitalization and ICU transfers for the BL-ASCT

Hospitalization for the BL-ASCT lasted a mean of 18.5 days (range 11–36 days). Out of 70 BL-ASCT events, there were six ICU transfers (11.7%), three of which were due to cardiovascular complications and one due to CNS bleeding. Mean number of days spent in the ICU was 0.34 (range 0–13).

### Mortality and TRM

There was 1 death out of 70 transplant events prior to day +30. This was a patient with a history of CNS germ cell tumor with persistent disease in the pineal gland area prior to transplant who succumbed to intracranial hemorrhage which was considered TRM. The second death event before day 100 was in a patient with multiple myeloma who contracted H1N1 influenza before day +30 post-SCT, developed ARDS, sepsis and died of multiorgan failure despite maximum supportive care. This death was considered to be TRM. Subsequently, no deaths were considered TRM. Overall, 100 day mortality was 2.9% out of 70 transplant events. Among the additional six patients who died after day +100, five patients died of disease progression, while one patient died of unknown cause close to 1 year post-SCT (cause unclear due to the lack of medical records). BL-ASCT TRM was 2.9%. One year mortality for the whole BL-ASCT cohort was 11.4%.

### Survival

**Fig. 1:**
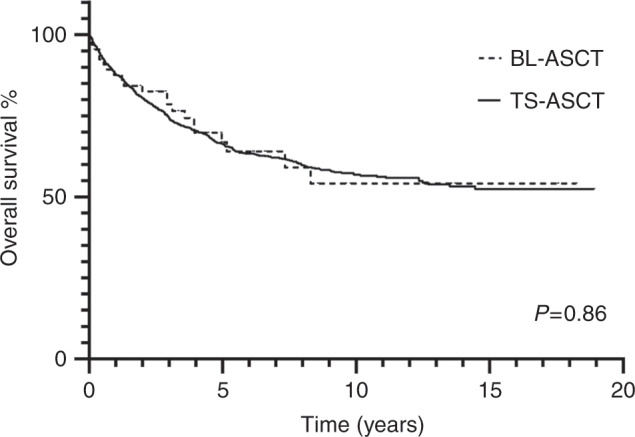
BL-ASCT and TS-ASCT patient Kaplan–Meier survival curve.

With a median follow-up of 63 months, 45 BL-ASCT patients are alive. The posttransplant OS was 87.9 % at 1 year for all BL-ASCT cases. There was no mortality prior to 1 year post transplant for those who received their second BL-ASCT. 30 day, 100 day, 1 year, and 5 year posttransplant OS did not differ between the BL-ASCT and TS-ASCT groups as illustrated in Fig. 1. Median OS was not reached for either group with a median follow-up of 63 months in the BL-ASCT group and 90 months for the TS-ASCT group.

Of the 70 BL-ASCT transplant cases, four cases are second transplants for the patients who had first BL-ASCT at our institution. None of the second transplant cases experienced mortality prior to 1 year post transplant. Out of 70, 68 BL-ASCT cases were alive at 100 days (97%) post-SCT and 62 out of 70 BL-ASCT cases were alive at 1 year (88.6%) post-SCT.

In addition, posttransplant survival between BL-AST and TS-AST, stratified by subgroups, such as age categories (<40 years, 40–59 years and ≥60 years), sex (male and female), ethnicity (Caucasian, Hispanic and African American), and diagnosis (multiple myeloma, non-Hodgkin’s lymphoma, Hodgkin lymphoma, and acute leukemia), did not show any significant difference.

## Discussion

ASCT improved OS and PFS in multiple malignancies, including relapsed lymphomas and multiple myeloma, and is a standard of care for the treatment of relapsed aggressive B cell lymphomas and Hodgkin lymphoma. Very few transplant centers in the United States and world-wide offer BL-ASCT.

This study supports the concept that BL-ASCT can be performed safely without significantly increased risk of TRM. In our study, there were only 2 cases of TRM out of 70 transplant cases with overall BL-ASCT TRM at 2.9%, which is <8% TRM in bloodless transplants reported by Ballen et al. and comparable with 2–4% TRM in TS-ASCT [2, 7, 14, 15]. Joseph et al. reported the lowest 0% TRM in BL-ASCT for multiple myeloma [7].

Supraventricular arrhythmia is a common complication of traditional autologous SCT. In 6 out of 70 (8.6%) BL-ASCT transplants, patients experienced cardiac complications manifested as arrhythmias. We do not believe there was a clinically significant increase in the incidence of arrhythmias in BL-ASCT cases as compared with previously described arrhythmia incidence in TS-ASCT. Analysis of 983 TS-ASCT cases in Nebraska demonstrated 9.4% incidence of arrhythmias [16]. In our study, arrhythmias were not associated with the degree of anemia in BL-ASCT patients, as the mean nadir hemoglobin nadir was identical at 8.7 g/dL in patients who experienced arrhythmias and those who did not. Based on our data, it does not appear that the arrhythmic complications in BL-ASCT group were related to the degree of anemia.

A platelet level of <10 × 10^9^/L makes bleeding one of the most feared complications in BL-ASCT [13, 14]. In our study, there were four episodes of bleeding in 70 transplant cases with a WHO grade > 2 and eight episodes of WHO grade 1–2 bleeding. In most cases, bleeding resolved promptly with medical management. Only one of the four higher grade bleeding patients had to be transferred to ICU due to hemorrhage. The second patient with higher grade bleeding had multiple medical issues requiring ICU transfer. Joseph et al. reported no bleeding-associated mortality in their series of 24 BL-ASCT cases, while Ballen et al. reports two cases of high-grade bleeding in 26 BL-ASCT patients [7, 14]. Based on our data, bleeding risk appears to be clinically acceptable to perform BL-ASCT transplants. In our study, most bleeding episodes happened during the time when the patients had platelet nadir below 5 × 10^9^/L, which is consistent with previously reported data by Ford et al. where no bleeding episodes occurred in BL-ASCT patients with platelets > 5 × 10^9^/L [13]. It is important to point out that at our institution as well as in all published BL-ASCT protocols, aminocaproic acid was administered to patients undergoing BL-ASCT when the platelet level decreased below 10 × 10^9^/L. Platelet threshold for the initiation of aminocaproic acid as well as aminocaproic acid dose vary between institutions [7, 8, 10, 13, 14]. There are no studies comparing the incidence and severity of bleeding with or without aminocaproic acid in the setting of BL-ASCT. In a prospective study, Wandt et al. demonstrated that TS-ASCT patients can be managed safely with therapeutic platelet transfusions without routine aminocaproic acid utilization [17]. Similarly, a randomized, open-label multicenter trial of prophylactic versus therapeutic platelet transfusions in patients with hematologic cancers demonstrated similar rates of bleeding in the two study groups among ASCT patients, and aminocaproic acid prophylaxis was not routinely used [18]. We support the updated 2017 ASCO guideline for the TS-ASCT which, allows experienced centers to use therapeutic platelet transfusion with the first sign of bleeding in adult patients as the majority of transplant-related bleeding episodes are nonlife threatening and resolve with supportive care even with the platelet transfusion-free approach [19]. The use of aminocaproic acid in therapeutic platelet TS-ASCT remains upon the discretion of the treating physician.

To date, there is no sufficient evidence to support the use of thrombopoietin mimetics in conventional or BL-ASCT. Several studies including our analysis report no bleeding-associated mortality in BL-ASCT performed without routine or emergent use of thrombopoietin mimetics [7, 15]. Coltoff et al. administered romiplostim prior to BL-ASCT to two patients who subsequently did not meet indications for transfusions and did not experience any bleeding. Al-Nawakil et al. administered romiplostim to 10 out of 13 patients on BL-ASCT protocol with mean duration of thrombocytopenia of <10 × 10^9^/L at 4.5 days [20]. In contrast, in our study, the median number of days with platelet count <10 × 10^9^/L was 3 without thrombopoietin mimetic use. Based on the current safety profile of BL-ASCT and due to the paucity of data regarding thrombopoietin mimetic use in ASCT we do not support routine use of thrombopoietin mimetics in conventional or BL-ASCT.

The utility and feasibility of red blood cell substitutes is a topic frequently addressed by patients who do not accept blood transfusions and their physicians. Unfortunately, none of the blood substitutes are approved by the US Food and Drug Administration. Synthetic oxygen transporters currently in development include perfluorocarbons and human or bovine hemoglobin oxygen carriers (HBOC). HBOCs can temporarily supply hemoglobin for oxygen delivery in critical patients with profound but reversible anemia. Hemoglobin based Oxygen Carrier HBOC-201 (Hemopure) is available for compassionate use for life-threatening anemia for patients where allogeneic blood transfusion is not an option. There are multiple case reports describing HBOC-201 use in JWs with critical anemia due to trauma, surgical complications, or acute medical illness. HBOC consistently provides increases in hemoglobin allowing time to perform additional measures for patient stabilization [20–23]. There are no reports of HBOC or perfluorocarbon use in stem cell transplantation. Compassionate use of HBOC-201 should be considered for critically ill JW with severe transient anemia undergoing stem cell transplantation where a temporary increase in tissue oxygen delivery would provide opportunity for other life-saving measures.

In our series, the patient with a history of CNS germ cell tumor with persistent disease in the pineal gland area prior to BL-ASCT succumbed to an intracranial hemorrhage early post-BL-ASCT. Ballen et al. report death from the intracranial bleed in a patient with active medulloblastoma [14]. We suggest exercising caution in performing BL-ASCT in patients with active CNS malignancy or a history of solid CNS malignancy.

In our study, OS did not differ between the patients who underwent BL-ASCT and the patients who underwent TS-ASCT for the same indications. Joseph et al. from the Emory University reported 24 multiple myeloma patients who underwent BL-ASCT with OS comparable with the matched cohort of TS-ASCT [7]. Therefore, all appropriate ASCT candidates who do not accept blood transfusions should have access to BL-ASCT.

The length of stay for the BL-ASCT as well as TS-ASCT varies between the studies. The median length of stay for the BL-ASCT patients in our study was 18 days (range 11–36 days), which is longer than 15 days (range 12–24 days) in BL-ASCT for the myeloma indication reported by Joseph et al., but comparable with 19 days (range 5–35 days) in BL-ASCT reported by Ford et al. For the TS-ASCT, Ford et al. reported average length of stay of 17 days (range 6–76 days) and Joseph et al. reported 16 days (range 12–29) [7, 13]. Based on these data, transplant centers should not expect clinically significant increased length of stay for BL-ASCT cases.

A limitation of this study is that this is a retrospective chart review and certain data points, such as disease status, could not be assessed to report PFS. The supportive care measures for the BL-ASCT were chosen empirically based on the theoretical benefit and were not studied in randomized controlled trials.

The Pew Research Center reports that JWs make up 0.8% of the US population at an ~2.5 million people, making an encounter with JW patients who need ASCT a common occurrence [24]. In our experience, JW patient referral to autologous transplant is frequently delayed as there is no widespread knowledge in the community and university practices that ASCT can be performed safely without significant increase in TRM in appropriate candidates. JWs have to travel long distances in order to obtain ASCT consultation and frequently have to undergo BL-ASCT far from their home. Complicated logistics of the BL-ASCT increases administrative burden on the referring oncologists and transplant institutions and causes financial and emotional stress to the transplant patients. Based on our data, we encourage community oncologists to promptly refer JW patients for transplant evaluation when ASCT is indicated. We encourage all transplant centers to embark on starting BL-ASCT programs.
